# Intraoperative tracheal reconstruction with bovine pericardial patch following iatrogenic rupture

**DOI:** 10.1186/1754-9493-2-4

**Published:** 2008-02-20

**Authors:** Nikolaos Barbetakis, Georgios Samanidis, Dimitrios Paliouras, Christos Lafaras, Theodoros Bischiniotis, Christodoulos Tsilikas

**Affiliations:** 1 Thoracic Surgery Department, Theagenio Cancer Hospital, A. Simeonidi 2, Thessaloniki, 54007, Greece; 2 Cardiology Department, Theagenio Cancer Hospital, A. Simeonidi 2, Thessaloniki, 54007, Greece

## Abstract

**Introduction:**

Iatrogenic injuries of the membranous trachea have become increasingly common and may trigger a cascade of immediate life-threatening complications.

**Case presentation:**

A case of a 48-year-old man with an iatrogenic membranous tracheal wall rupture after double-lumen intubation during Ivor Lewis esophagogastrectomy is presented. Tracheal injury was successfully managed surgically with the use of bovine pericardial patch and reinforcement with the gastric conduit which was moved toward the posterior wall of the membranous trachea sealing the wall laceration.

**Conclusion:**

Our technique was proved to be safe, effective and not technically demanding. Early recognition with prompt surgery is the gold standard of managing such cases, although small tears can be managed conservatively.

## Introduction

Iatrogenic injuries of the membranous trachea are rare but potentially lethal and can complicate procedures such as endotracheal intubation, mediastinoscopy, percutaneous tracheostomy and excision of neoplasms with mediastinal involvement. Statistically women are more predisposed to this than men [1]. Further risk factors include poor medical condition, short stature, use of steroids, previous chemotherapy and mediastinal radiotherapy. A case of a 48-year-old man with an iatrogenic membranous tracheal wall rupture after double-lumen intubation during Ivor Lewis esophagogastrectomy is presented. Tracheal injury was successfully managed surgically with the use of bovine pericardial patch and reinforcement with the gastric conduit which was moved toward the posterior wall of the membranous trachea sealing the wall laceration.

## Case presentation

A 48-year-old man was admitted to our department with the diagnosis of an adenocarcinoma of the gastroesophageal junction. Preoperative staging was negative for distant metastases. Endoscopic ultrasound findings confirmed the presence of a locally advanced tumor (type I – Siewert classification). It was an adenocarcinoma of the distal esophagus arising from an area with intestinal metaplasia and infiltrating the esophagogastric junction from above. As a consequence preoperative synchronous chemoradiotherapy was proposed. The scheme was consisted of fluorouracil (15 mg/kg of body weight) daily for five days and cisplatin (75 mg/m2 of body surface area) on day 7 and a course of radiotherapy (40 Gy administered in 15 fractions, anterior-posterior mediastinal fields) over a a three week period, beginning concurrently with the first course of chemotherapy. After the completion of chemoradiotherapy, the stage of the disease was reevaluated (CT scans of the abdomen, chest, pelvis, upper gastrointestinal endoscopy and ultrasound). Restaging was completed with no evidence of distant disease and surgical removal of the primary tumor and lymphadenectomy was proposed. The patient was eligible for an Ivor Lewis esophagogastrectomy.

He was intubated with a left-sided 37-F double-lumen endotracheal tube by a specialist anesthesiologist experienced in cardiothoracic surgery anesthesia. The technique includes the following points. The tube was held with the bronchial curve concave anteriorly. As the tip was passed through the larynx, the tube was rotated 90 degrees to direct the endobronchial part to the intended side. The tube was then connected to the breathing circuit via a double catheter mount.

**Figure 1:**
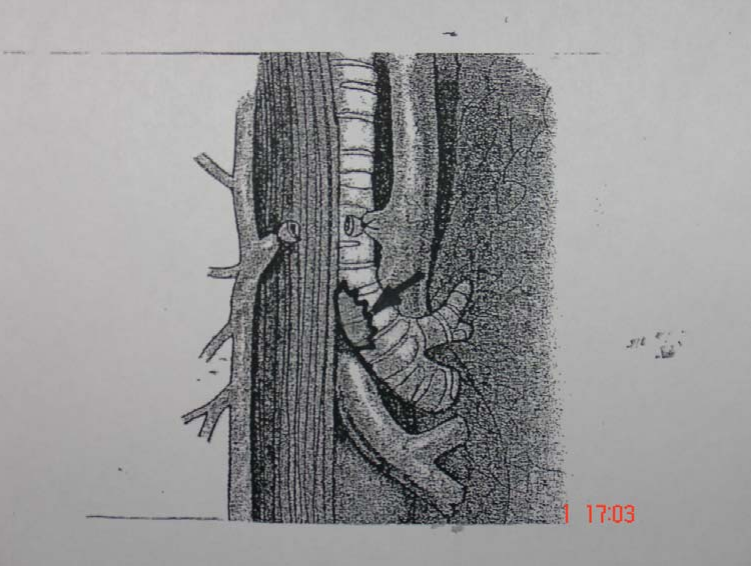
Oval-shaped laceration (arrow) of membranous trachea extended to 1 cm above the carina.

The abdominal part of the operation was uneventful. During the second phase and while the intrathoracic esophagus was being mobilized a hissing noise was heard and a careful inspection revealed part of the double-lumen tube and the inflated tracheal cuff protruding through the ruptured membranous tracheal wall into the operating field. This resulted in an approximately 3 × 1 cm oval-shaped laceration of membranous trachea, extending to 1 cm above the carina (Figure 1). The ventilation and oxygenation of the patient could now only be continued by occlusion of the tear by the surgeon's finger and subsequently by a surgical gauze swab. Our first attempt was primary interrupted suturing which failed and resulted in enlargement of the laceration probably due to the previous chemoradiotherapy. Tracheal repair was then performed by tailoring and suturing a bovine pericardial patch, using a running Vicryl 4-0 suture. This led to control of the leakage and normal ventilation was re-instituted. The main operation was completed by performing a classical Ivor Lewis esophagogastrectomy with a 2-field lymphadenectomy. Esophagogastric continuity was restored with an end-to-end anastomosis using an EEA 25 mm circular stapling device.

**Figure 2:**
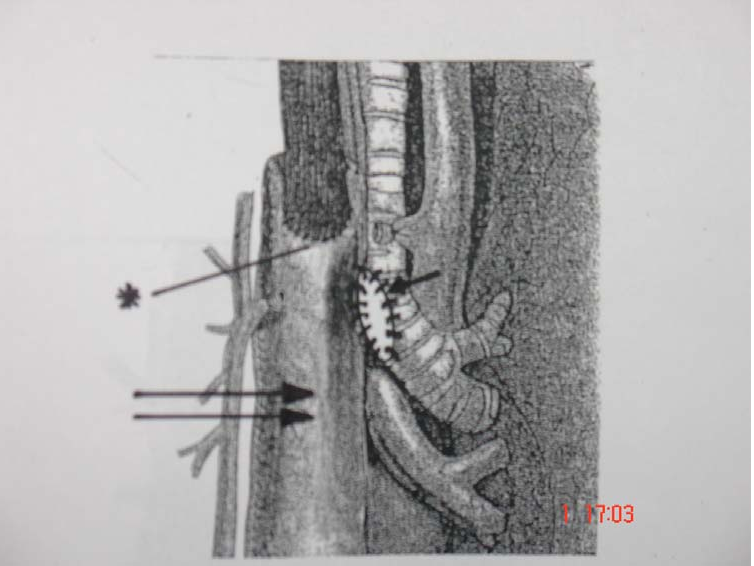
The gastric conduit was moved toward the posterior wall of the membranous trachea by means of single interrupted nonabsorbable 3-0 prolene sutures which were placed between the stomach (double arrow) and the lowest edge of the rigid tracheal rings (arrow) (asterisk – esophagogastric anastomosis).

In order to reinforce the tracheal repair at the end of the operation, the gastric conduit was moved toward the posterior wall of the membranous trachea. Single interrupted nonabsorbable 3-0 prolene sutures were placed between the stomach and the lowest edge of the rigid tracheal rings (Figure 2). This manipulation moved the gastroesophageal conduit into contact with the pericardial patch on the posterior wall of the membranous trachea, sealing the wall laceration. In order to check the effectiveness of our technique the pleural cavity was filled with normal saline and the ventilation pressure was raised to 30 cm H2O. A careful inspection for air leakage was negative. Two thoracic drains were placed as usually and the patient was transferred to the intensive care unit intubated and mechanically ventilated. A no 7.5 single lumen endotracheal tube was substituted for the double-lumen tube and was advanced bronchoscopically to just above the carina. The patient was extubated on the second postoperative day and had an uneventful recovery. Nutrition and subsequent airway management were in line with our usual protocols. Chest tubes were removed on 9^th ^postoperative day, two days after the patient started to feed orally. He was discharged home on 14^th ^postoperative day without any respiratory or swallowing problems.

## Discussion

Tracheal rupture after endotracheal intubation is a rare entity. Most retrospective reviews focus on elective intubations by experienced anesthesiologists and show an incidence of rutpture of less than 1% [2]. There are multiple factors leading to injury. Operator errors (multiple attempts, inexperienced phycisians), equipment selection (inappropriate use of stylets, cuff overinflation, malposition of the tube, incorrect tube size), patients' movements (abrupt movements, excessive coughing) and medical factors (steroid or radiation-weakened membranes, chronic obstructive pulmonary disease, tracheomalacia) contribute to the problem [3]. Other risk factors include age>50 years, short stature, obesity, double lumen tubes and percutaneous tracheostomy [3]. Women are more frequently affected by an iatrogenic tracheobronchial rupture. Certainly, their small body size and therefore the risk of placing the tube too distally in a short trachea and an incorrect tube size might be responsible. Additionally, a vulnerable and weak trachea is often suspected in women[4].

Iatrogenic tracheal rupture involves almost exclusively the membranous trachea [5]. Early recognition with prompt surgery is the gold standard of managing such cases, although small tears can be managed conservatively [6]. In general tracheobronchial ruptures of the middle and lower third are operated on through a right and in rare exceptional cases, a left thoracotomy [7]. The approach to injuries in the upper third of the trachea is the left cervical side. Angelillo-Mackinlay proposes a transcervical access in the sense of a mediastinotomy for injuries to the distal trachea, which is, nevertheless, fairly controversial, due to the creation of additional trauma (longitudinal tracheotomy)[8]. For the repair, patients should be intubated using a double-lumen tube. Single lumen tube with position in the contralateral main bronchus or high-frequency jet ventilation can be used alternatively. However, disadvantages of the jet ventilation technique are the tendency to produce carbon dioxide retention and the danger of blood aspiration into the bronchial system. After limited lateral and posterior paratracheal dissection, the injury is repaired by interrupted or running absorbable suture, sometimes covered with mediastinal fat or pleural flap and/or by fibrin glue [9]. Direct suture of a tracheal rupture in a patient previously treated with chemoradiotherapy is not an option as this was proved in our case and resulted in enlargement of the laceration. Our technique adopted here, was based on the competence of the gastric wall to sustain the tension against the tracheal rings and furthermore to push the bovine pericardial patch toward the posterior wall, sealing the tear. The above mentioned technique is proposed for surgical management of tracheal wall lacerations as it proved safe, effective and not technically demanding. The use of an endotracheal stent could be proposed but the urgent situation (almost impossible ventilation and oxygenation) led us to resolve the problem surgically. However, the employment of a covered expandable stent should be considered for the treatment of a tracheal or bronchial tear. The development of these stents represents an effective, direct and less invasive means of sealing tracheal tears [10]. Stent deployment commits the patient to continued outpatient surveillance to monitor for complications. Although uncommon these include granulation tissue formation, stent migration, halitosis and recurrent respiratory tract infections [10].

Iatrogenic tracheobronchial ruptures are mainly caused by emergency intubations [1]. Percutaneous dilational tracheostomies and double-lumen intubations do not show higher signs of complications compared with single-lumen intubations or conventional tracheotomies, if they are accompanied by verification through bronchoscopy [1]. Patients without any respiratory failure and a small tracheobronchial rupture may undergo conservative treatment. They must be checked by repeated bronchoscopies to detect granulation tissue and relevant tracheal stenosis. Early surgical treatment of the latter must be the therapy of choice.

## Competing interests

The author(s) declare that they have no competing interests.

## Authors' contributions

NB, GS, DP, CL and TB took part in the care of the patient and and contributed equally in carrying out the medical literature. CT participated in the care of the patient and had the supervision of this report. All authors approved the final manuscript.

## Consent

Written informed consent was obtained from the patient for publication of this case report and accompanying images. A copy of the written consent is available for review by the Editor-in-Chief of this journal.
